# Applying Self-Regulated Learning and Self-Determination Theory to Optimize the Performance of a Concert Cellist

**DOI:** 10.3389/fpsyg.2020.00385

**Published:** 2020-03-06

**Authors:** Guadalupe López-Íñiguez, Gary E. McPherson

**Affiliations:** ^1^Sibelius Academy, University of the Arts Helsinki, Helsinki, Finland; ^2^Melbourne Conservatorium of Music, The University of Melbourne, Melbourne, VIC, Australia

**Keywords:** artistic research, intra-individual, learning identity, metacognition, mixed-methods, practice, self-determination theory, self-regulated learning

## Abstract

The professional practice of classical music performers has been better understood and enhanced across the last two decades through research aimed at tailoring rehearsing strategies that support the development of a sense of self as an agentic and proactive learner. One approach focuses on helping students make use of various tools that can enhance their learning, particularly in terms of what they do, feel and think when practicing and performing music. This study expands literature on expertise development by embracing the idea that this line of research would benefit from additional studies where the researcher forms part of the research process as an active participant who generates data, especially when these researchers are “members” of the social world they study, and therefore have insider knowledge. Thus, this case study is focused on the first author, a professional cellist who is also a researcher in the educational psychology of music, as the only participant. It extends current research by providing a detailed longitudinal mapping of a professional cellist’s preparation across nine profiled concerts in five countries of classical-romantic repertoire and a commercial recording that resulted from 100 weeks of dedicated practice. Anonymous feedback from the audiences and interviews with an expert musician who followed the concerts and the CD recording was also collected. For the data analysis, traditional psychometric measurements were applied to test the internal consistency of the time series data as well as the relationship between variables. In addition, the application of Leximancer analysis of the self-reflections allowed the researchers to probe self-regulated learning (SRL) and self-determination theory (SDT) processes in ways that uniquely mapped, over time, her differing motivations to perform at a high level. Specifically, we report that the cellist’s psychological needs and her motivational resources changed across time within the social context of performing music publicly, and that the various self-regulatory processes she drew upon impacted (both positively and negatively) on her ongoing actions, thoughts and feelings. Implications of the study are relevant for all forms of expertise development research, and especially for understandings about the nature of skill development in the context of learning to perform demanding literature in music.

## Introduction

One way researchers have begun to make significant improvements to the professional practice of classical music performers is through personalized rehearsing strategies that support the development of a sense of self as an agentic, autonomous, and metacognitive learner. The focus of these types of investigations is on improving the various self-regulated learning (SRL) strategies associated with the behaviors (actions), cognition (thoughts), and affect (feelings) musicians employ when practicing and performing music, and that support a proactive engagement with music making and music learning (McPherson and Zimmerman, 2011; McPherson et al., 2019).

Within music, an important theme in SRL research has focused on helping students apply various tools that can enhance their learning (e.g., McPherson et al., 2018, 2019). These self-regulation behaviors that musicians adopt during their performance have been identified as crucial for improving both performance and learning, as evidenced by Pike (2017) in her case study of a piano student, and with a larger number of advanced, skillful music students who have been shown to engage with this type of activity autonomously, especially when practicing their instrument (Papageorgi et al., 2010).

A major component of self-regulation is metacognition, with the cognitive and motivational component of this construct being seen as crucial to optimizing musicians’ practice (Hallam, 2001; Nielsen, 2011) and performance (Concina, 2019). In music, experts have been shown to demonstrate higher levels of metacognitive competence as they self-regulate themselves while learning (Concina, 2019). In addition, self-regulation behaviors appear to be particularly favored by advanced musicians (Araújo, 2016).

**FIGURE 1:**
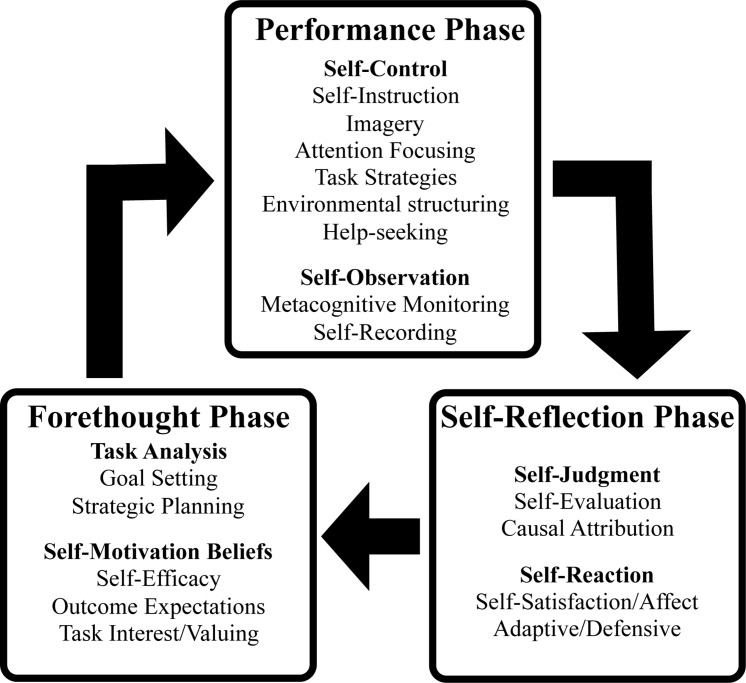
Phases and sub-processes of self-regulated learning (from Zimmerman and Campillo, 2003, p. 239).

Regardless of the level of expertise, the ability to self-direct one’s learning involves three cyclical phases (see Figure 1) in which an individual analyses a task they are about to complete (forethought), employs various self-control and self-observational skills to focus their attention when completing the task (performance), and then makes self-judgments and experiences self-reactions concerning the quality of their efforts in preparation for the next practice session or performance (self-reflection; Zimmerman and Moylan, 2009; Cleary and Zimmerman, 2011; Cleary et al., 2012; McPherson et al., 2018).

Self-regulation has been linked to the efficiency of music practice and seen as vital in the development of musical expertise (Miksza, 2011; McPherson et al., 2019), especially in the context that expert musicians have typically undertaken thousands of hours of practice to refine their craft (Ericsson et al., 1993). The importance of such experience in connection to the development of expert musical performance has been studied by tracking the behaviors of music students during childhood and adolescence while practicing their instruments (e.g., Gabrielsson, 1999; McPherson and Davidson, 2002; Miksza, 2006, 2007, 2011; Palmer, 2013), as well as the practice efficiency and dedication of classical musicians (e.g., Gruson, 1988; Sloboda et al., 1996; Lehmann and Ericsson, 1998; Chaffin et al., 2002; Jørgensen, 2002; Williamon, 2004; Altenmüller et al., 2006; Lehmann and Jørgensen, 2012; Jørgensen and Hallam, 2016). Much of this research has been influenced by the “deliberate practice” framework of Ericsson et al. (1993), which asserts that focused and attentive forms of practice that are cognitively demanding lead to the best outcomes for skill development (see Platz et al., 2014 for meta-analysis). Other lines of research examine how much of the variance is accounted for by practice alone (McNamara et al., 2014; Bonneville-Roussy and Bouffard, 2015) and the motivational dimensions of novice to expert musician’s engagement when practicing (Nielsen, 2011).

**FIGURE 2:**
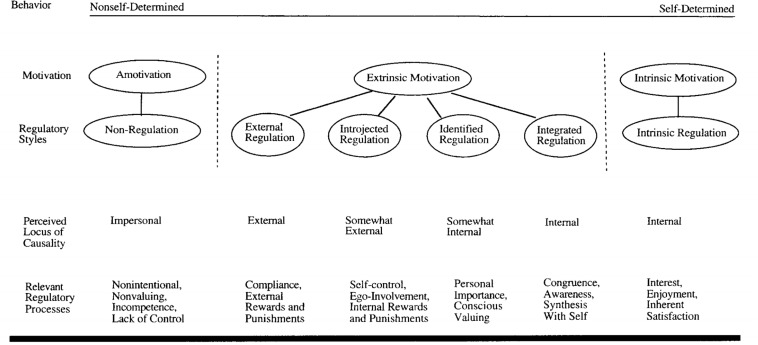
The self-determination continuum showing types of motivation with their regulatory styles, loci of causality, and corresponding processes (from Ryan and Deci, 2000, p. 72).

Recently, music students’ motivation (Evans and Bonneville-Roussy, 2016), as well as their deliberate practice approach (Hatfield, 2017) have been studied using self-determination theory. Ryan and Deci’s (2017) self-determination theory (SDT) provides a unified theoretical approach to the holistic study of motivation in musicians (Evans, 2015), including the social, cognitive, affective and behavioral factors mentioned above. Self-determination theory plays a crucial role in understanding the six types of motivation, from amotivation to self-determined or intrinsic motivation as the extreme poles (see Figure 2), particularly over extended periods of time (e.g., Georgiadis et al., 2006; Reinboth and Duda, 2006; Sheldon and Krieger, 2007; Alivernini and Lucidi, 2011; Jang et al., 2012).

The various forms of motivation within the SDT model are related to the satisfaction of three fundamental human psychological needs: autonomy (volition, personal choices and decisions), competence (self-efficacy) and relatedness (social connectedness) (Ryan et al., 1995; Hagger et al., 2006). Thus, it is understood that the more these psychological needs are satisfied, the more self-determined autonomous motivation (Deci and Ryan, 2002; Gagné and Deci, 2005; Hagger et al., 2006) and, on the contrary, the more they are thwarted, the more the individual will procrastinate, adopt defensive reactions, and end up feeling demotivated and/or helpless.

Researchers in music have highlighted the importance of intrinsic motivation to overcome difficulties when experiencing failure (e.g., McPherson, 2012). To date, little research has dealt with the intertwined dimensions of self-determination and self-regulation in relation to both practice and performance. Within one-to-one music studio settings (in depth reviews in Küpers et al., 2014; Evans, 2015) these studies have examined the relationship of SRL and SDT with well-being and participation (Creech et al., 2013; Krause et al., 2019), or the frequency and quality of practice in music students (Evans and Bonneville-Roussy, 2016; Schatt, 2018; Valenzuela et al., 2018).

### Rationale and Purpose of the Study

The purpose of this study was to explore the self-regulatory and self-determination processes of a female concert cellist across 100 weeks of rehearsal as she prepared for a series of concerts and the recording of a commercially available CD. The study sought to examine the professional cellist’s psychological needs satisfaction and various types of motivation in a longitudinal intra-mental process of self-regulation that would optimize the cellist’s performance across profiled concerts and a commercial recording. Key issues included how the musician regulated herself according to the psychological dimensions which underpin intentional self-regulation processes, and how her motivation aligned with her self-reported cognition, behavior, and affect within a social context.

In this regard, the study differs from previous single-case, long-term studies by other authors. For instance, the reports of cellist Tania Lisboa and her colleagues, whose series of research studies have provided invaluable information on musicians-researchers’ subjective experiences of learning music by heart, focused on technical, interpretive, structural and performative aspects such as tempo stability (e.g., Chaffin et al., 2010; Demos et al., 2016). These studies were carried out across public and practice performances with one movement of Bach’s No. 6 Suite for solo cello (ca. 5 min of music) through inconsistent/interrupted data collection procedures. Previously, Chaffin et al. (2002) had similarly studied tempo stability through preparation and performances of one movement of Bach’s Italian Concerto for piano over a period of 57 weeks. Another example of performance tempo variability research is the study by Lehmann and Ericsson (1998) where the authors used a period of 9 months to analyze the development of an advanced piano student from the sight-reading stage to a single public concert performance phase.

To expand on these studies, our study sought to offer new insights into the characteristics of music performance skills of professional classical musicians striving to improve their practice and performance by: (1) expanding the length of time, amount and difficulty of repertoire by including chamber musicianship instead of solo performances; (2) increasing the longitudinal span of data collection to 100 weeks; (3) using original period instruments with a historically informed approach to performance, thus making this study multidisciplinary through the combination of psychology and musicology; (4) employing a mixed methods approach in artistic research; (5) studying not only motoric and cognitive skills development, but also behavior and affect with a special emphasis on intersubjectivity; and (6) focusing on the relationship between SRL and SDT.

## Materials and Methods

A mixed-methods, concurrent triangulation design (see Creswell, 2013) was employed, framed in the art-based educational research (ABER) and creative analytical practices (CAP) to generate practice-based knowledge (for an overview, see Gouzouasis, 2019). This research approach embraces the view that significant inroads to increasing knowledge in this area may be achieved through studies in which researchers are active participants who generate data as a result of their membership of the social world being studied (e.g., Ellis and Bochner, 2000; Denzin, 2006). The advantages of employing this type of insider knowledge to reinforce research on expertise development is highlighted in recent publications that advocate the use of such mixed-methods approaches in order to “build a rich picture of phenomena exploring subtle social dynamics and interactions between individuals and employing organizations […] trying to understand what is happening, when, and why” (Yardley et al., 2020, p. 412).

A quasi-experimental approach (León and Montero, 2002) examined within-person change and variability through an intervention that consisted of making self-reflections and filling in surveys across concerts and a commercial recording. More specifically, the study examined whether and how such interventions (independent variable effects: concerts and diary interventions) affect the repeated measures of the dependent variable (basic psychological needs), in trajectories of time (pre-, during-, post-rehearsals) and in different stages (concerts, recording).

The self-reflective approach to this study was framed within Boud et al. (2013) three major elements of reflective thought processes of individuals: (1) *returning to the experience* immediately after the event has finished, to clarify missed details and perceptions; (2) *attending to feelings* to assess the affective/emotional impact of the event; and (3) *re-evaluating the experience* to integrate the new knowledge into one’s existing knowledge or repertory of behaviors.

This was complemented by a single-case descriptive approach to describe the intervention in the real-life context in which it occurred (Yin, 2003). For this, the researchers employed a visual inspection approach to make judgments about whether the independent variable was affecting the dependent variable based on level, trend, and latency (Fisch, 2001). This approach helped to reduce the limitations found in retrospective self-report measures that are frequently used in SRL studies (see McPherson et al., 2019).

### The Cellist

This single case study was focused on the first author, a professional cellist who is also a researcher in the educational psychology of music. As a female Spanish citizen in her early thirties, now residing in Finland, she is an active professional musician with a Master’s Degree in Cello Performance who specializes in solo recitals and chamber music concerts on period instruments, and whose concerts and recordings have been critically acclaimed internationally. In addition, the participant-researcher has obtained a Ph.D. in Psychology, having specialized in social constructivism (psychological school) in music teaching and learning.

### Procedures

The study investigated the intra-individual change and variability in intentional self-regulation through the researcher’s learning of canonic Classical-Romantic era works for the instrumentation combination of cello and piano, as she optimized her performances over a period of 100 weeks (from 29/7/2016 to 19/10/2018) across 9 invited profiled concerts in 5 countries (see below) and a commercial CD recording (*Complete Mendelssohn Piano and Cello Works on Period Instruments*, Alba Records, ABCD434, 2018). During this time, she learnt and performed the following pieces by Beethoven and Mendelssohn—all considered artistically demanding because of their stylistic and technical demands. This repertoire comprised a total of 150 min of music.

#### Repertoire

Ludwig van Beethoven (1770–1827):

• Sonata Op. 5. No.1 in F major (1796)
• Sonata Op. 5. No.2 in G minor (1796)
• 12 Variations WoO45 in G major (1796)
• 12 Variations Op. 66 in F major (1796)
• 7 Variations WoO46 in E-flat major (1801)

Felix Mendelssohn Bartholdy (1809–1847):

• Variations Concertantes Op. 17 in D major (1829)
• Albumblatt (Assai Tranquilo) in B minor (1835)
• Sonata Op. 45. N.1 in B flat major (1838)
• Sonata Op. 58. N.2 in D major (1843)
• Lied ohne Worte Op. 109 in D major (1845)

#### Concerts

(1) October 2016. Sellosali Concert Series, Espoo (FI)
(2) November 2016. Theatrum Kloostri Ait, Tallin (EST)
(3) May 2017. Nordic Historical Keyboard Festival, Kuopio (FI)
(4) September 2017. Aino Ackté Festival, Helsinki (FI)
(5) November 2017. New Pavillion Concert Series, Kauniainen (FI)
(6) April 2018. Beethoven Research Center, San José (US)
(7) July 2018. Piano Salon Christophori, Berlin (GE)
(8) August 2018. BRQ Vantaa Festival, Vantaa (FI)
(9) October 2018. Moscow Philharmonic Society Small Hall, Moscow (RU).

#### Forms of Data

For the purpose of the analyses reported here, forethought data included:

Printed sheets including questions with dichotomous and Likert-type scales related to the types of motivation described previously. The sheets included descriptions and examples of the six types of motivations to cue the cellist’s self-reports before practicing, as follows:

• Intrinsic Motivation (enjoyment, pleasure, internal, process oriented): *Today, I am studying because I think the task/goal/piece is interesting and I like/enjoy the learning process* (Scores from 1 to 10—less to more)
• External Regulation (external pressures, operant conditioning): *Today, I am studying because I want to do well on the next concert/rehearsal with pianist, or because I want to gain trait/rewards/success or avoid punishment* (Scores from 1 to 10—less to more)
• Integrated Regulation (coherent personal behaviors to achieve the goal): *Today, I can remain calm when facing learning difficulties because I can rely on my abilities and adopt the necessary behaviors* (Options: yes/no/partly)
• Identified Regulation (value of the outcome, learning what needed): *Today, I have a fierce desire to overcome obstacles or challenges that lie within today’s task/goal/piece no matter what* (Options: yes/no/partly)
• Introjected Regulation (internal pressures, ego): *Today, I feel I would need feedback/approval from a professional or from other people because I am stuck/under pressure with the task/goal/piece and do not know what to do* (Options: yes/no/partly)
• Amotivation (completely unmotivated): *Today, I have no interest/intention to practice and will do nothing to change this situation* (Options: yes/no).

All inventories were adapted from the theoretical models by the researchers. Validity and reliability of the measurement techniques were provided by: (1) defining preliminary questions and the types of possible answers in relation to the six types of motivations for the instrumental music domain with the support of an expert in self-regulation studies who possesses expertise in developing and validating questionnaires and rubrics within the field of learning sciences; (2) piloting the questions during 5 consecutive practicing days approximately 3 months before submitting the research proposal to carry out this project (which was subsequently funded); (3) refining independently subtle aspects in the questions and answers that arose during the pilot of the measurements; and (4) using the theoretical model to add descriptions and examples independently.

For the self-reflection phase, data consisted of:

• The same questions, including dichotomous and Likert-type scales, as in the forethought phase (though adapted by using past tense as they were referring to the immediate finished practice session) so that the cellist could self-report about her motivation after practicing.
• Personal self-reflections in English and Spanish in which the cellist described her actions, thoughts and feelings during the particular practicing session and overall week connected to it.
• Feedback from the audience and concert/CD critics (see Supplementary Appendix 2).
• Comments from an independent expert (see Supplementary Appendix 3) who listened to all concerts either live or via high quality video in order to provide an independent external assessment of the overall artistic value and quality of each performance and the resultant CD recording.

#### Processing of Data

The above data was collected approximately once a week, on average, and both the exact practicing days and the selected repertoire per concert are provided in Supplementary Appendix 1. The amount of accumulated practice time for each of the reported days in this study ranged from 40 to 100 min per session (*M* = 65 min). The cellist did not gather information on accumulated practice during other practicing days where there were no diaries involved. However, her total practice estimation during the whole artistic project was approximately five times more, as she kept a regular practicing routine that ensures she practiced at least 6 days a week.

Data preparation was undertaken from source document (printed sheets) to machine readable form (computer files) then edited or translated as necessary prior to the analyses. Source documents were stored in a locked file cabinet. Computer files were saved in the internal hard disk of a computer with password and as a backup in an external hard disk with the password kept within a locked cabinet. Data integrity was measured in terms of accuracy, timeliness and relevance, as it was transcribed immediately and systematically after each data collection point. At the time this article was published, data was not available in any open data repository.

#### Performance Approach

As a cellist, the first author specializes in historically informed performance practice (HIPP), and her performances were informed by a musicological approach that included visiting sites where the original and copyist manuscripts of the music to be performed and other miscellanea related to the composers were located. These included the Bodelian Library in Oxford, the Staatsbibliothek zu Berlin, the Heinrich Heine Institute of Düsseldorf, the Biblioteka Jagiellonska in Kracow, the Mendelssohn-Haus in Leipzig, and the American Research Center in San José, California (for more information, see López-Íñiguez, 2019a, b).

#### Cello and Bows

A cello by Claude Pieray (built in Paris, 1725) with an instrumental set-up inspired by examples from the early 19th century, and classical-transitional bows by François Tourte (built in Paris, 1800) and by André Klaassen (built in Zutphen, 2015) were used for all rehearsals and performances. The first two tools have been loaned to the performer for lifetime by a private donor, whilst the third is her own property.

#### Pianists and Keyboards

For this research project, the cellist was accompanied by three different professional pianists in both modern and period keyboards, who performed on romantic pianos and fortepianos (the detailed list of these particular instruments can also be found in Supplementary Appendix 1).

### Analyses

We performed a linear regression on the overall dependent variable *motivation* against both independent variables *concerts* (understanding concert as a continuous variable on a scale from 1 to 10 study sessions) and *intervention* (pretest versus posttest).

In order to quantify and display the conceptual structure of the open questions in the diaries, and explore conceptual features over time, we also used the content analysis tool LEXIMANCER (2018). This approach was employed because it reduces the bias that can potentially occur when researchers code or categorize their own data, even after using reliability procedures involving other experts, and to ensure through a complex network analysis that the researchers do not become fixated on certain concepts to the detriment of others (see section “Post-Practice Self-Reflections, Leximancer Analysis” for further details).

## Results

The following sections include descriptive statistics for the Likert and dichotomous questions regarding the five types of motivation^1^, as well as linear regression results. We also include a section that provides the Leximancer analysis of the self-reflections across concerts.

### Five Types of Motivation

**FIGURE 3:**
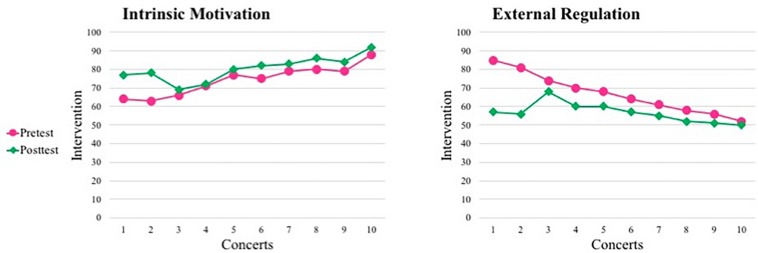
Visual tendency for intrinsic motivation and external regulation. NB: *Pretest* refers to the piloting of the measures across 5 consecutive practicing days before commencing the intervention; *Posttest* refers to the period of intervention data collection.

Figure 3 shows the visual tendency according to the scores (from 1 to 10 points) assigned to the questions based on Likert-type rating scales for intrinsic and external regulation across concerts (10 practicing sessions per concert). The scores can be observed in Supplementary Appendix 4.

**FIGURE 4:**
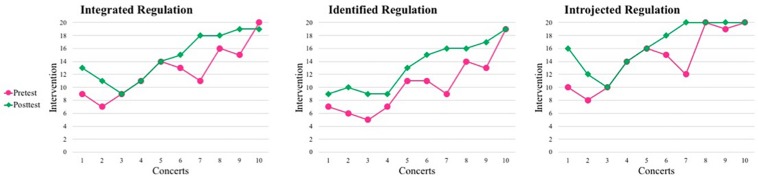
Visual tendency for integrated, identified, and introjected regulation.

Figure 4 shows the visual tendency according to the dichotomous questions (yes/no/partly) for the subtypes of extrinsic motivation, namely integrated-identified-introjected regulations across concerts (10 practicing sessions per concert). The scores are shown in Supplementary Appendix 4.

#### Effect of Intervention and Concert on Motivation

A linear regression on the same items was employed to test significant differences in the motivation items pre- and post-intervention and after all concerts. For each motivation and regulation type, the pretest and posttest scores were plotted against concert, to display the scores and help assess the pretest *versus* posttest difference. A linear regression was performed for each, with motivation being the dependent variable and the independent variables being concerts (understanding concert as a continuous variable on a scale from 1 to 10 study sessions) and intervention (pretest *versus* posttest). Regression coefficients were estimated along with their associated standard errors, and *P*-values calculated to test the significance of the coefficients (see Table 1).

**TABLE 1:** Effect of intervention and concert on motivation.

	***p* Intervention**	**β Intervention**	**SE Intervention**	***p* Concert**	**β Concert**	**SE Concert**
Intrinsic Motivation	0.000	−0.630	0.148	0.000	0.214	0.026
Integrated Regulation	0.054	−0.210	0.108	0.000	0.111	0.019
Identified Regulation	0.013	−0.290	0.116	0.000	0.120	0.020
Introjected Regulation	0.065	−0.200	0.108	0.000	0.118	0.019
External Regulation	0.000	1.030	0.140	0.000	–0.236	0.024

As shown in Figure 3, there was a clear increase of intrinsic motivation throughout all concerts while external regulation decreased. According to all concerts and the 10 rehearsals sessions within each concert, a linear regression found significant differences in these two types of motivation pre- and post-intervention and after all concerts (see Table 1).

Figure 4 showed that integrated, identified and introjected regulations increased markedly during the project. A linear regression on these three same items found significant differences pre- and post-intervention and after all concerts only for identified regulation what comes to study sessions’ progress within a concert (see Table 1).

In order to understand how these particular aspects of motivation shaped the cellist’s practice sessions and the underlying reasons for her self-determined motivation, the following section includes an analysis of the musician’s post-practice self-reflections where she describes her actions, thoughts and feelings.

### Post-practice Self-Reflections, Leximancer Analysis

In order to explore conceptual features over time in the self-reflections after each concert (including 10 practice sessions each), we applied a lexicometrical analysis—with the help of an expert user of this software—employing the standard granularity threshold of 100% for visible concepts and 50% for theme size (concepts), and a basic conglomerate of related words (thesaurus). For example, using this system *practiced* and *practicing* would go under the concept of *practice*; with *player, performer, cellist* and *musician* under the concept of *musician*. Leximancer is:

“[a] method for transforming lexical co-occurrence information from natural language into semantic patterns in an unsupervised manner. It employs two stages of co-occurrence information extraction—semantic and relational—using a different algorithm for each stage. The algorithms used are statistical, but they employ non-linear dynamics and machine learning” (Smith and Humphreys, 2006, p. 29).

Leximancer software uses a combination of techniques such as Bayesian statistics that records segments of text to identify semantic concepts. Then, it associates those concepts to a thesaurus of words that provide each concept with their definitional properties.

**FIGURE 5:**
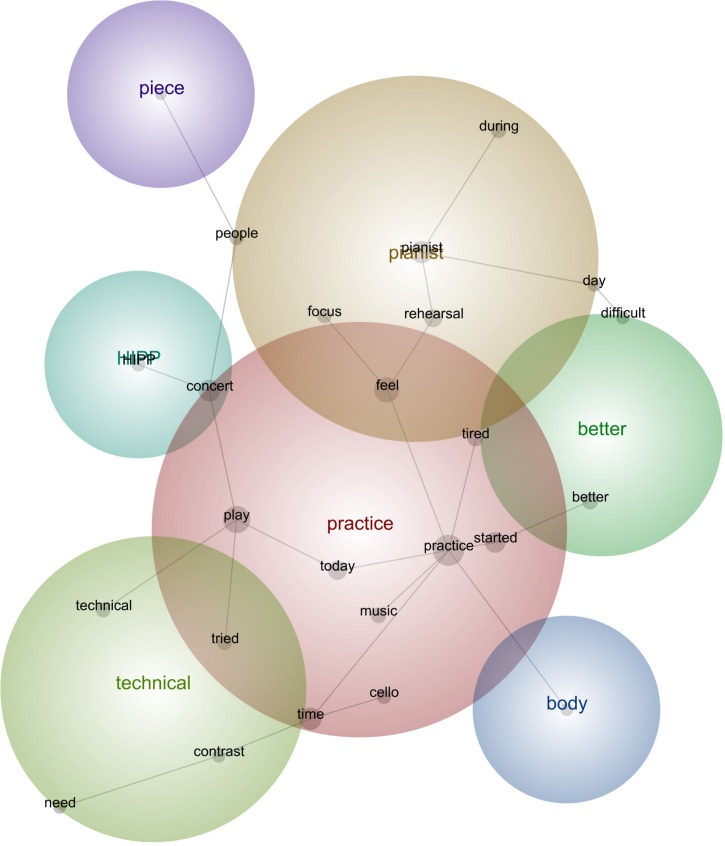
Leximancer concept map for Concert 1.

**FIGURE 6:**
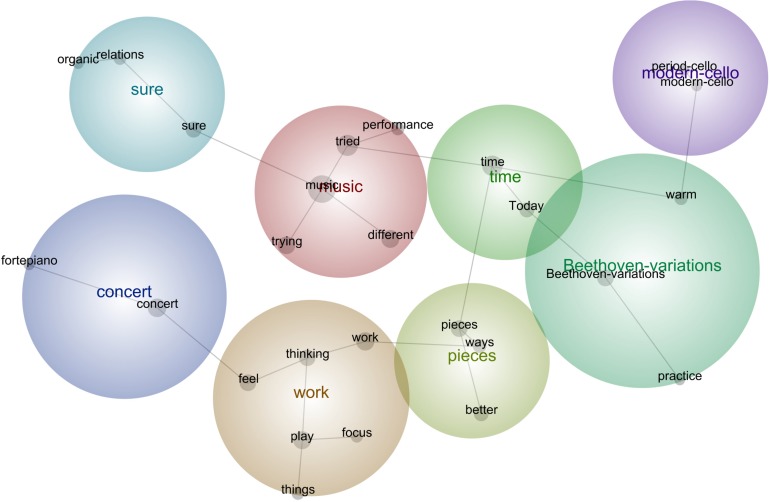
Leximancer concept map for Concert 2.

**FIGURE 7:**
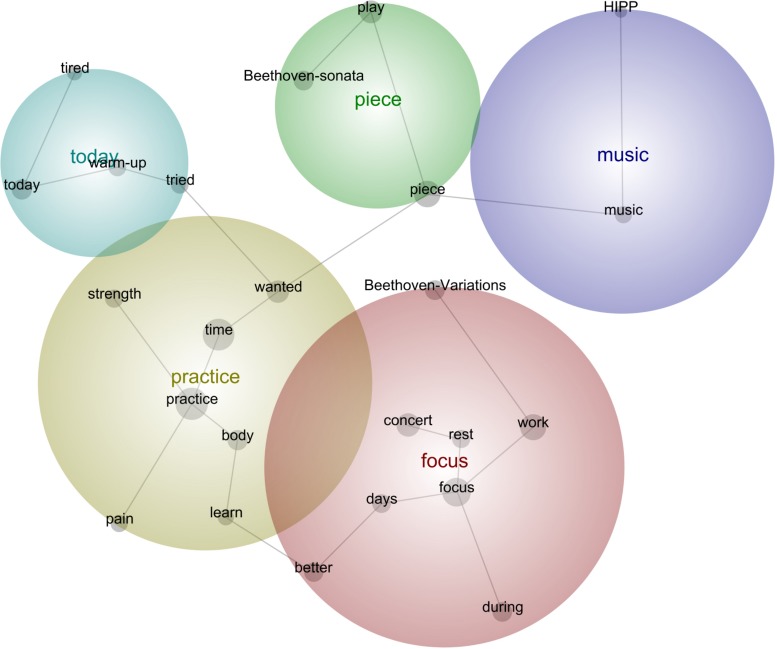
Leximancer concept map for Concert 3.

**FIGURE 8:**
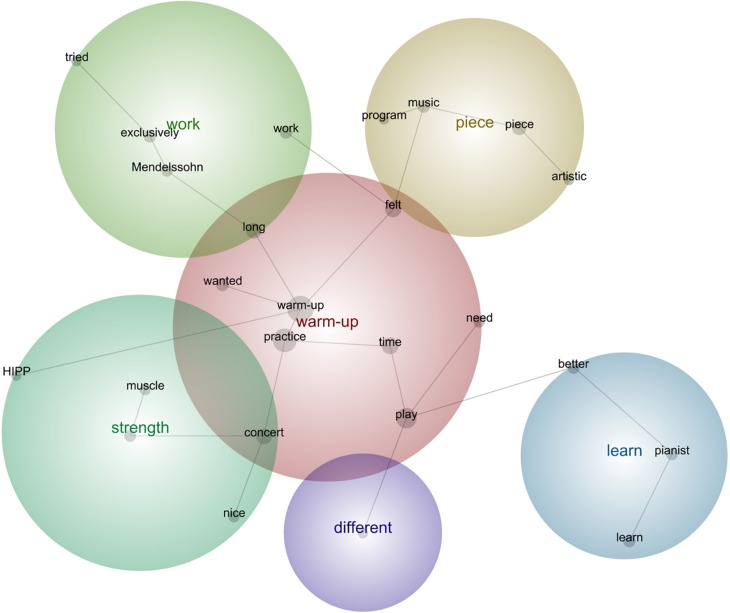
Leximancer concept map for Concert 4.

**FIGURE 9:**
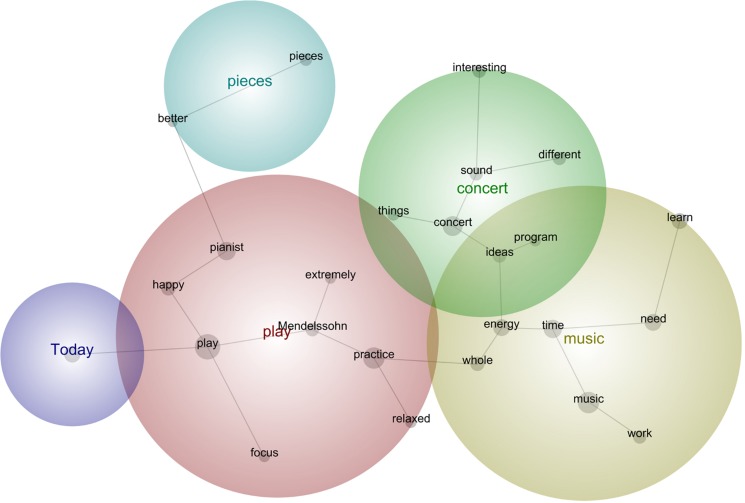
Leximancer concept map for Concert 5.

**FIGURE 10:**
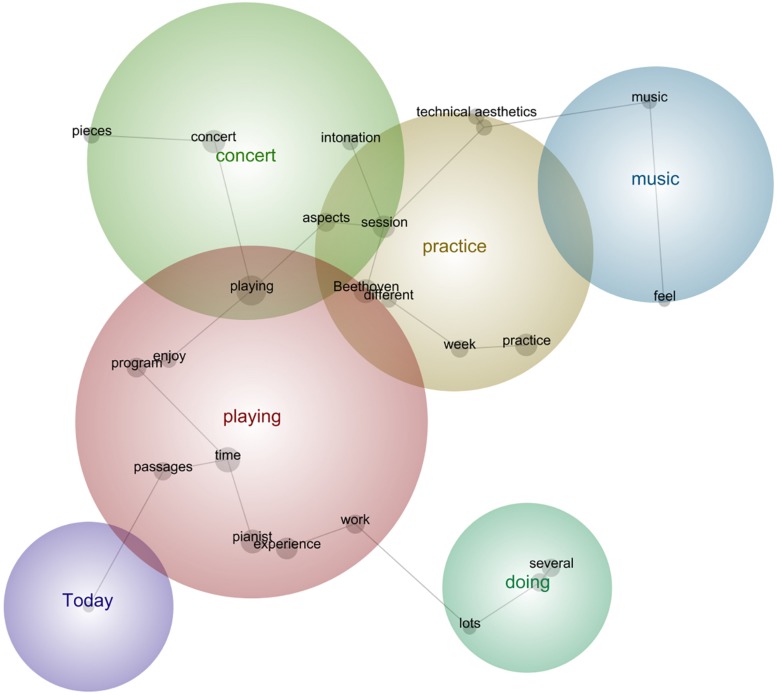
Leximancer concept map for Concert 6.

**FIGURE 11:**
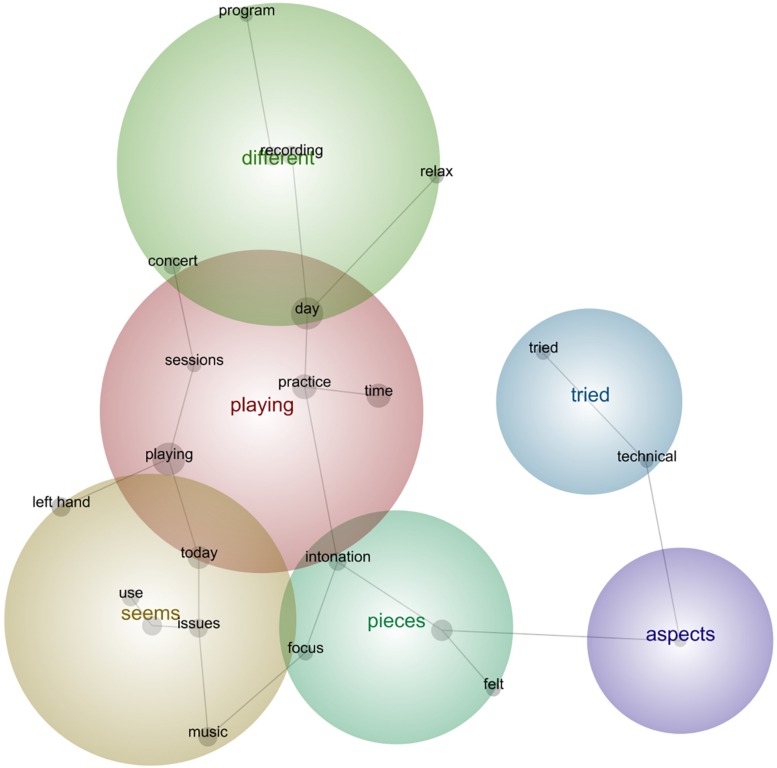
Leximancer concept map for Concert 7.

**FIGURE 12:**
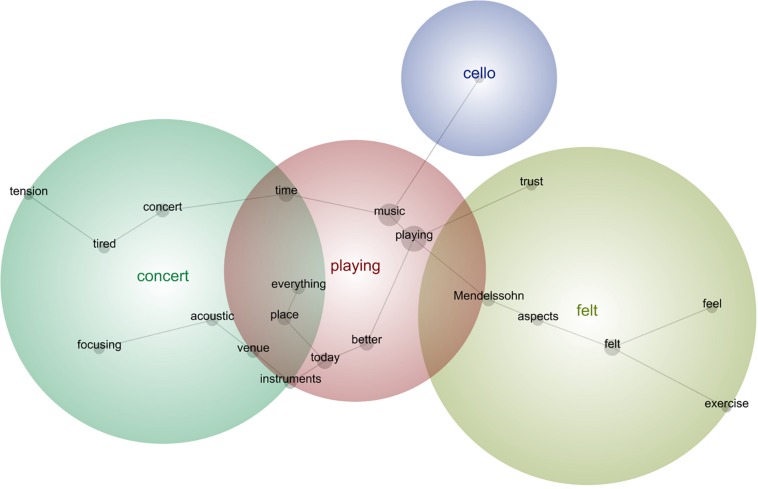
Leximancer concept map for Concert 8.

**FIGURE 13:**
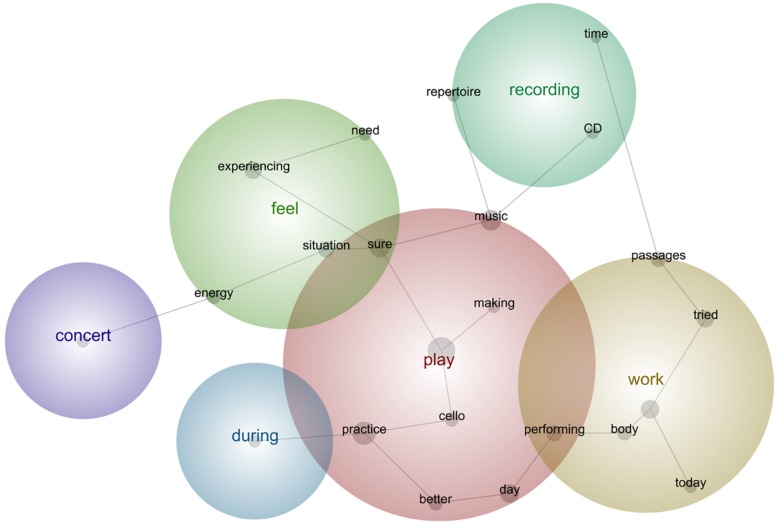
Leximancer concept map for Concert 9.

In the next sub-sections, we followed the common practice in lexicometrical studies (e.g., Bécue-Bertaut, 2010) by using bold for emphasis of the main semantic concepts identified during the analysis as per their relative frequency, strength, and prominence. Provided below are the prototypical phrases that support the main concepts selected by this tool per concert (see Figures 5–13). The selected phrases are introduced in order (from earlier to later diaries within one concert). We have included a narrative description of the aspects featured in all stages at the end of this section.

#### Concert 1

“I have to prepare my body **better** before **practicing**. **Difficult** things will be **better** tomorrow.”

“I would need more knowledge about **HIPP** [Historically Informed Performance Practice] to frame my **practice** particularly toward misunderstood Mendelssohn’s **pieces**.”

“The worst part is to be mentally ready to play with the **pianist**, so that I **feel** strong on stage. She is too patronizing **during rehearsals**. I need **better people** to support me in this project.”

“I tried to widen the emotional range of each variation and the character **contrast** between each variation. I **need** to work on **technical** issues to **play** the **music** by Beethoven more fluently. I **need** more **time** in general.”

“I was extremely nervous in the **concert**. I invited too many important **people** and some musicians-colleagues, and that plus the attitude of my **pianist during** all the **rehearsals** preparing for this **concert** made me **feel** crazy, so I could not **focus** well for the practicing time prior to the concert, it was a bit of nonsense, besides my energy was really low, my hands **tired**.”

#### Concert 2

“**Today** I was going through both **Beethoven-Variations**, **thinking** of how to improve the phrasing, to make it more natural and flexible, more singing, but also more fluid because even if I can **play** all the notes right, it doesn’t carry well. I was **trying** to **play** them with more relaxed body, with a **different** bow, changing some fingerings and bowings for the less physically demanding solutions, and **things** like that, but it is not as it should be.”

“I was **trying** to **work** on the sudden virtuoso places of the **Beethoven-Variations**, because you jump from **playing** the simplest basso continuo line to the most virtuoso passage, so I was **trying** to have breaks and jump suddenly into the difficult passages, and that was useful.”

“The next **concert** is radio-broadcasted, so I have to perfect my tone quality, even if it usually is good, but make all changes of positions and bowings smoother and cleaner, so I was doing a bit of that too, because it needs a very special atmosphere, so I **tried different ways** to express the emotions behind the **music.”**

“I have been **thinking** a lot about the **ways** to **feel** more secure with the pianist, and the only way is to avoid talking about anything, and just **play** and **focus** on the **piece**s, so I was **working** on a **better** rehearsal plan, and the **things** that I would like to improve, as well as more chamber **musicianship**, more connection between us, not just **playing** together.”

“**Today** I **focused** on connecting the movements of the Beethoven-Sonata in a way that the **piece** is a whole, and not a package of **different** movements. I was making **sure** the tempi **relations** were **organic**, and I was **trying** to understand the logic behind them, because for the first **concert** I just did what everyone does, but this **time** I **tried** to look beyond and see what the tempo giusto for this **music** could be, I **tried different** types of tempo **relations**, and also of speed for each movement, till I found some nice solutions which I marked with the pencil on the score. I want to make **sure** during the next **times** that these tempo **relations** seem **organic** most of the **times**, so that I make them somehow the final version for my **performances**.”

“I was **today** comparing the **performance** of these Beethoven-Sonata and **Beethoven-Variations** with the **modern-cello** and the **period-cello**, and it was very revelatory. I **tried** to perform the **pieces** as a modern cellist would nowadays, and then compare that with the HIPP approach, and it led to very interesting performative conclusions.”

“I was **thinking** about how did the bowings **feel** in the previous **concert**, so I **tried** to make a compromise between the perfect style and a comfortable enough **performance**, physically speaking.”

“**Today** I decided to **think** positively about myself and my **playing**, and about the possibility to succeed in the coming **concert**.”

#### Concert 3

“I have **tried** to **focus** on the new **Beethoven-Sonata**, this will be the first **time** I **play** this one, and I **wanted** to dedicate extra **time** for this **piece** this week. It feels a little easier than the other **Beethoven-Sonata**, yet there are a few tricky passages, so I was **working** on them. It is also a very idiomatic **piece**, so I quickly found easier solutions for fingerings and bowings which follow the same **HIPP** logic as the ones I have used in the other **pieces** by Beethoven, so this was a lot faster process than before, as I am **learning** the language for this **music**.”

“I will repeat the same kind of **work** for some **days**, to get used to it and **learn** the **pieces better** and **better** by reflecting on the physical feelings when I do such **work**, so that the **body** memorizes the movements and feelings for the **concert**.”

“I was **practicing today** before my rehearsal with the pianist, and I **wanted** to save some energy because every **time** I meet this person I run out of energy, and I need a lot of mental **strength** to go through the rehearsals. Others’ limitations are not my limitations, so I was **focusing** meticulously on what I am doing during the practice, like in a laboratory.”

“I **want** this **piece** to sound perfect, because I love it, so I was a little stressed out in the exercises I did with some passages, **wanting** quick results, so I have to be more patient and **learn** progressively. I cannot demand quick results if I really **want** to know the **piece** well and own it. I’m **learning** to **focus better during** my sessions, these diaries seem to help, so I’m more reflexive when I choose what to do, and particularly how not to procrastinate or think about something else while I **practice**. I have set my phone off **during** my **practice**, and that is really good too, and I have been doing more Yoga.”

“I have less than three weeks before the **concert**, so it should be ok to have a **day** off and just sleep well and **rest**, because I see no point to **practice** with this lack of **focus** and **body pain,** and **tiredness** after the whole academic year and traveling all the **time**, yet, I **tried** my best to **practice focusing** on what I **wanted** to **learn** and develop, to maintain the muscle engagement, so that my **strength** develops progressively.”

#### Concert 4

“As I have enough **time** before the next **concert** and I’m on holiday, I can focus to **practice** specific passages without **playing** the **pieces** through and **try** to truly develop and improve the places that **need** more **time**, so I was focusing **exclusively** to those passages that are still in progress. Really good **work**.”

“Today I **felt** I **need** to **play** the **Mendelssohn-Sonata** really well and memorize it a bit **better**.”

“Today I **wanted** to compare the **HIPP** approaches to the Beethoven-Sonata and the **Mendelssohn**-Sonata. I **wanted** to analyze and **learn** what performative and technical aspects are similar and **different**, so that I am more aware of these aspects when I perform, and make them even more clear for the audience, and for the **pianist**.”

“Today I was determined to check the density of the piano parts, which also gave me a good idea of where I could do even more **artistic** and creative things, and when there is enough space for improvising in **different** ways with the tempo, the rubato, the rhythmic asynchrony, the portamenti, etc.”

“I did a great **warm** up today, the child is at school and I don’t have teaching yet, so I had plenty of **time** to **practice**, with enough breaks, some tea **time**, some stretching, some relaxation, a great and **long warm** up, and breathing and technical exercises. Then, I went into the most demanding passages in each **piece** for this **program**, and I really **felt** I have progressed and **learnt** a great deal about this **music**, yet the **work** is not finished yet, so I must keep on to honor the **music**.”

“I **try** to save some energy for the **concert**, so I was thinking how to share the **muscle strength** and mental energy between Beethoven and **Mendelssohn** in the **concert**, so that I am not exhausted at the end, as this is the **long**e**st concert** we will have **played** so far for this project, so this is important.”

#### Concert 5

“I did a great practicing session because I **focused** exclusively to see the development of **Mendelssohn**’s styles across the **whole concert program**, all his **music** for cello and piano, and that was a really **interesting** and inspiring strategy which kept me **working** despite being exhausted and with a little neck tension and back and shoulder plate pain. I think I will **work** on this style **thing** during the next days, as I feel I **need** to **learn** much more about the shuttle differences in the complete **music**.”

“**Today** I was **extremely happy** and motivated to **practice** because I changed my **pianist** for a much proficient **pianist**, and also a more **relaxed** person, I had the first rehearsal with her **today**, and the chemistry was immediate, besides I could **play** some difficult stuff in an easier way because the **pianist** was able to **play** her parts without problem and understood the tempi and character in a similar way as I do. I am really **happy**, motivated, and feeling more and more confident. I basically enjoyed **playing** the **pieces** through without the mental turmoil I have experienced so far. I felt relieved and wanted to only **focus** on positive **playing** experiences in my study **today**, to recover from the negative past.”

“I have **played** the **Mendelssohn**-Sonatas so much that **today** I wanted to **focus** exclusively on the smaller **Mendelssohn**-Albumblatt and **Mendelssohn**-Romance. It was lovely to **work** on them as they are so **different** in the character. They are not technically impossible, so I tried to be as polished as possible, making them perfect technically and stylistically, without losing the passion and character, or being **uninteresting**. They are so short that I **played** them through several **times**, and I am to do the same in the next days because these are the **pieces** that the audience know and love the most, but I also want them to sound good for myself!”

“**Today** I **worked** on the **different** characters and intonation issues according to the keys and harmony of the **Mendelssohn**-Sonatas. They are really **different** and they both take a lot of **energy**, so I was trying to build **interesting** moments to keep the tension and emotional engagement of the audience through them. I just love working on these **pieces**.”

“**Today** I was video-recording my **practice**, which I should do more often. I have been so bored practicing these **pieces** over and over again, even if I love them, that I did not know what else I could do. I watched the video of the run through the **whole concert program** and got several **ideas** for improvement.”

“**Today** I had a good day because I got big funding for the **Mendelssohn** CD recording, which gave me a lot in encouragement and eagerness to **practice** and dream about the **whole thing**. I was really motivated and **happy**, and I did a great job despite being tired and again with wrist and neck pain.”

“I have to save my **energy** for the **concert**, as this **player** is stronger than the previous one and I **need** a bigger **sound**, therefore more **energy** investment.”

#### Concert 6

“I could see that I had done a great job learning these **pieces**, and many of the **aspects** were still there, though I selected a few difficult expressive **passages** to bring out more singing and emotional output. I did a great warm up, and I had plenty of **time** to stretch, drink water, and have a tea break, so the **session** felt ideal, and I identified things I want to improve further, such as the clearer articulation of the left hand and some **intonation** issues. I’m also excited for this **program** because I will **play** in the United States for the first **time**, in a very special **Beethoven** place with great fortepianos and another **pianist**, so that was helping me to focus and **enjoy** the practicing **session**, because I am motivated and happy, **feeling** lucky and privileged to do this **work**.”

“I tried to **play** the later repertoire by **Beethoven** in order to make even more clear the characters and **technical aesthetics** of the earlier **music**. I spent **several** hours **doing** this because it was a rather reflective **work**, and I needed **lots** of **time** to think about what I was **doing** and if it made any sense, but it did and I learnt a lot. I **enjoy** much more **playing** the earlier **pieces**, and I was thinking about the **aspects** I want to mention in my **program** notes to the audience.”

“I did some **intonation work** with the tuning machine, and some Starker, and I moved to the **Beethoven**-Sonatas, just trying to **play** the **pieces** by heart, which went rather well. I **feel** I own the **pieces** now, and that the long break was actually needed to avoid artistic and physical burnout.”

“**Today** I recorded my **practice**, to see how the clarity of my **playing** is, and how clean the virtuoso **passages** sound. I wanted to prepare well before I go to the United States and meet the new **pianist**, so that I can **enjoy** the rehearsals as much as possible. I was visualizing a positive relationship with the **pianist**, and a successful **concert experience** while **playing** the **program** through, while trying to breath and relax the body right before the virtuoso **passages**, so that I am really in control.”

#### Concert 7

“I have done some mental **work** today. I have decided to **practice** the **concert** in a **different** way than the **recording sessions**, as the audience is a **different** thing than a microphone, and that **playing** 90 min recital needs a **different** energy optimization than an 8-h **recording session** with snacks and indoors all **day**. So, I just **focused** on getting back to the **program**.”

“I think I need a period of working with the **left hand** enough, because that is my weakest part, and it needs still some muscle strength and confidence.”

“I **practiced** the simpler Mendelssohn-**Pieces**, **trying** to reach a great sound quality and expression, without any **technical** mistake, yet with lots of singing qualities, until I was pleased.”

“**Today** I recorded my **practice**, just without **playing** the **concert** through, only a regular **practice**, because I’m so bored of this **program**, that I wanted to see what I could do to inspire myself, and toward what kind of direction I could move. I noticed that I **play** too much without enough breaks, even if it **seems** that I am stopping, so I should be even more conscious on how I schedule my **practicing sessions**, probably using a timer, though it is demanding for me as I am quite a free person what comes to **time use**.”

“**Today** I just **played** through the **pieces**, work slowly on a few passages that gave me a headache before, and video-recorded the **session**, which was useful as I noticed some **issues** with my right arm position and **use**, which **seem**s to be less **relaxed** than I wish, even though I had not **felt** any problem while **playing**. So, I was **focusing** to put the shoulder a bit lower, and breath a bit more, and all started to sound so much better that I loved the **practicing session**.”

#### Concert 8

“I had no eagerness to **play** the same **music** over and over again today, it gets **tired** when you have practiced it for so long, that musical ideas do not appear anymore. So, instead of doing the usual work, I was reading the score in peace without the **instrument**, just making sure I know every single annotation by the composer, and I also spent some **time playing** the **music** with the modern **cello**, to remember the physical differences, and the artistic options I have with a period **cello**. I think I need a little break for a couple of days and **play** something else to avoid hating this **music**.”

“**Today**, I did not **play** the **Mendelssohn** repertoire at all. Instead, I did the longest warm up ever, and I **played** some etudes and **exercises** from relevant treatises, which **felt** good and refreshing. I just wanted to make sure I still love my **cello** after this long project.”

“**Today** I did two run throughs of the program. One **focusing** to relax the body and breath, and the other one **focusing** to exaggerate the character of the pieces. This was a wonderful **exercise** because, technically, I **feel** ready, so I enjoyed myself a lot.”

“I decided to spend the day **playing everything** slowly in my mind first, in the order of the **concert**. It was really exhausting, but super useful, as when I started to **play** the **cello**, many things **felt** really fluent, I should do this **exercise** more often as it is less physically demanding, yet one learns a lot. I also thought about all the journey with the learning of **Mendelssohn**’s **music**, and how far I have reached, yet how much more one could learn and how many stylistic **aspects** are unclear for performers, so I tried to **focus** to the ones that I really **feel** secure and confident about, and **play** them really clear.”

“**Today** we practiced in the **concert venue** both individually and together, we had a good **time** there as this **venue** has the perfect **acoustic** for this kind of **music**, intimate and helping the balance and overtones, so I really enjoyed myself **focusing** on the harmonics and resonance of the **instrument**s, and how easy it was to **play** in tune with the fortepiano, that **everything** was in the right **place.** I think I should go for **better acoustic place**s because this is a crucial **aspect** to keep happy.”

#### Concert 9

“I have got some physical strength and my fingers and arms **feel** stronger, so the practicing takes less **energy**.”

“**Today**, I **played** the program through three times. I wanted to **make sure** that I am able to do so, therefore I will **feel** really strong and powerful when I only have to **play** it once **during** the **concert**.”

“I know myself **better** according to my mood, mental and physical **situation**, **energy** levels, focus, **situation** of the instrument ant the people I play with, and I am not **sure** what to write anymore, except that I am faster solving any issue that has to be fixed with this **repertoire**.”

“**Today** I focused on only one thing that has kept me happy **during** the last days, which is to enjoy practicing as much as possible, without any specific **work** plan beyond **playing** musically and in a confident manner, with a relaxed **body** and a calm mind. Just **performing cello music** for myself.”

“I am not sure what else I could **try** out at this point after so many **concerts** and the **recording** preparation, somehow, I **feel** I need new **repertoire** and style, and have a good holiday.”

#### Description of the Aspects Featured in All Stages

Concert 1 involved aspects such as body control and technical development to tackle part of the repertoire, setting up a routine to practice, starting the first HIPP and emotional connections of the music with the instrument playing, and realizing the external pressure due to the first professional concert situation and the negative connection with the pianist.

In Concert 2 the cellist became more strategical in her practice toward refining the emotions and technical clarity in the pieces, particularly working toward a radio broadcast for the second concert. She endeavored to establish a positive mindset after reflecting on how the previous concert went and adopting more complex strategies for the HIPP style through the use of different instruments. Continuity of the music as a holistic conception of performance was also worked out in relation to phrasing and tempi. Dealing with the pianist was thought out carefully by means of preparing mentally prior to rehearsals and avoiding verbal communication in order to survive the situation.

Concert 3 dealt with the application of different solutions to new part of the repertoire based on the previous concerts and pieces in connection to muscle memory and identifying problems from the beginning to avoid losing time, which led to quicker learning. Mental preparation before rehearsals with the pianist to avoid low energy and negative feelings was also considered. Conditions for practicing such as avoiding the use of electronic devices, preparing the body better through physical exercise, resting when exhausted, and becoming more reflexive when preparing the study sessions were crucial in this stage.

Concert 4 involved working with more selected passages, memorizing the music, and comparing the different technical aesthetics for Beethoven and Mendelssohn, while keeping the pianist and audience in mind to convey these aspects clearly. In addition, a more creative and improvisatory focus than in previous concerts stages was adopted, including the organizing of practicing sessions in a better fashion (breaks, warming up), including mental and body awareness to cope with the concert demands.

Concert 5 included work on more subtle differences within a composer’s style, refining the music making, saving energy for the concert, and enjoying having a more supportive and proficient pianist than previously. Focusing on the cellist’s own enjoyment of the music playing while practicing, thinking about communicating them to the audience and honoring the composer were also considered. Using video-camera to provide feedback on how to improve further was a useful strategy, and external funding proved to make a difference in the cellist’s overall mood at this stage of the artistic project.

Concert 6 dealt with the emotional engagement with the music and perfecting even more the technical aspects in selected passages, becoming more reflective and organized with the practice, and preparing mentally in a positive way for a concert with another pianist.

Concert 7 aimed to build more muscle strength through selected exercises to cope with a more demanding concert and the recording, putting more focus on expression than on technical aspects, and thinking about how to improve the practice sessions’ overall structure for time optimization. Awareness of body tensions through video-camera was also considered.

For Concert 8, reading the scores and mental practice, plus a variation of practicing strategies due to tiredness of practicing the same repertoire in similar ways with the instrument for so long were employed. Becoming more confident, enjoying the concert venue, and focusing on the performance were at the center of this preparation.

Concert 9 was about rejoicing practicing and preparing positively for the performance, whilst recognizing the development throughout the project in relation to body control and muscle strength, ownership of the repertoire and the instrument, and confidence as a performer who is also looking toward future artistic projects.

## Discussion

This study examined the self-regulatory and self-determined processes of a single professional cellist across a period of 100 weeks, as she prepared for performances and a commercial recording. It sought to understand how the cellist’s psychological needs and motivational resources changed across time, and how her behavior, cognition and affect within the social context of performing music publicly was aligned to her use of various self-regulatory processes that impacted (both positively and negatively) her ongoing actions, thoughts and feelings.

The cellist is a professional musician and concert cellist who was capable of undertaking this study as a single participant due to external funding to carry out an artistic research project of this kind, and also having the technical resources and knowledge to prepare the research design described above. She was capable of devising specific goals for her practice, and approaching tasks strategically, particularly in terms of assessing her future success and failure. She also possessed clear artistic career intentions that were evident throughout the project and especially during the performance that culminated in a commercial recording of the literature she prepared. Finally, her strong intrinsic motivational orientation and desired to be autonomous and personally competent formed the basis from which she was able to continuously monitor her practice and choose behaviors that would support her in achieving her goals, particularly by feeling and thinking more positively about herself and her own learning.

There are several factors that helped the cellist in her performance and learning optimization development across the study. On the one hand, there was a clear increase of intrinsic motivation throughout all concerts while external regulation decreased. According to all concerts and the 10 rehearsals sessions within each concert, significant differences were found in these two types of motivation pre- and post-intervention and after all concerts. The cellist explains this in connection to two main factors: (1) the change of pianist for a more supportive one and improving her overall conditions for the project (mental and physical work, better concert venues and instruments), and (2) the desire to become more autonomous, confident, and competent both technically and artistically. This shift toward more specific and intrinsically rewarding music objectives and focused practice as the project developed is consistent with reports from studies dealing with tertiary music students’ practice, where SRL instruction has been used to change traditional learning approaches (Miksza, 2015).

On the other hand, integrated, identified and introjected regulations increased markedly during the project. Significant differences were found as per each concert and all study sessions within a concert, whereas differences pre- and post-intervention and after all concerts were only found for identified regulation what comes to study sessions’ progress within a concert. The results concerning integrated and identified regulation align with the cellist’s continuous desire to learn the necessary skills to succeed in each concert according to her predetermined goals. At the same time, the overall increase in these types of regulations after each concert can be explained because the cellist wanted to avoid external disapproval and achieve her outcomes, even if sometimes she relied on internal pressure to achieve this due to the importance of the project for her and, for example, time and conditions constrains to make it happen—thus keeping a professional attitude for both rehearsals and performances as she focused her attention on preparing for quality concerts. Additionally, the feedback from critics, audience and the expert musician who attended the concerts—generally positive and encouraging—as well as the external funding received during the project, could be linked to the increase of, particularly, introjected regulation, as these narratives were regularly appearing while the cellist was rehearsing in the studio or performing publicly, and therefore extrinsically influencing her learning processes. However, as might be expected through extrinsic forms of motivation, their importance can be interpreted as less influential since they did not appear in the Leximancer analysis.

Overall, this continuous engagement in self-monitoring and self-observation of behavior across the period studied led to an increase in the cellist’s metacognitive ability to apply more progressively complex rehearsal strategies. For instance, as described in section “Description of the aspects featured in all stages,” the cellist monitored her learning by:

• *drawing together more complex connections among artistic and aesthetic ideas*: for example, from trying out the first HIPP ideas to being able to apply a selection of them after reflecting which ones better suited her style and the music;
• *using information in new situations*: for example, assessing how concerts went and how further improvements could be made in future concerts, in addition to reflecting on what comes to kinesthetic and muscle memory or dealing with external pressure and selecting a supporting pianist; and
• *attributing success and failure and planning tasks accordingly*: for example, from being less organized in the study and feeling constant musculoskeletal pain to creating regular habits for mental and physical practice.

She acknowledged that her constant responses on the printed sheets throughout the project cued her desired development as per a choreography of learning habits, strategies, and abilities that she had developed across her entire musical development (McPherson et al., 2019). This also shaped the way she pursued and refined her own identity as a learner (Coll and Falsafi, 2010)—a type of learning mindset that has recently been associated with musicians’ career success and enjoyment (López-Íñiguez and Bennett, 2020)—, in ways that allowed her to thrive motivationally (Ryan and Deci, 2002).

The cellist’s self-determined motivation was studied through a lexicometrical analysis of how relatedness, competence and autonomy acted to increase or decrease motivation by means of more progressive complexity in all stages of preparation. Prototypical phrases selected by the Leximancer software across concerts showed that during rehearsals, the cellist was constantly improving on (1) her technical and strategical learning processes, (2) her mental preparation before concerts, rehearsals with pianists and individual study sessions, (3) her body awareness and physical development as per muscle strength and energy; (4) the conditions that affected all stages, such as different pianists, venue acoustics, type of audience, broadcasting, funding and praise, and (5) her short- and long-term artistic goals.

Our analyses highlight strategies that the cellist adopted to optimize her learning and performance in preparation for concerts and a CD recording. Many of the aspects are also present across the entire life of a musician, such as striving for technical proficiency through progressive muscle development, employing a particular style to a certain repertoire, or working on mental preparation and memory processes. There are, however, a number of aspects inherent in this 100-week preparation cycle that the cellist has not previously experienced for other artistic projects. These include (1) understanding how emotional and professional support from peers is crucial for feeling competent and thriving, (2) working on more creative, stylistic, and improvisatory aspects of music making, particularly in terms of the canon repertoire, to help select better strategies for learning and performances fresh and unique, (3) using video-recording study sessions and rehearsals to better inform the path to a desired outcome, and (4) taking care of the body as a fundamental aspect in the life of a performing musician.

Systematically organizing her own thoughts, feelings and actions in connection to her learning and performance goals, helped the cellist to balance the quality and quantity of practice undertaken during the period of preparation. For instance, working on mental preparation and physical strength, changing her first pianist for a more supportive one, continuously focusing on improving technical and expressive-aesthetic aspects of the repertoire, and becoming more selective with her practicing strategies and the conditions of the study sessions leading up to a series of successful concerts and commercial recording (in line with Usher and Schunk, 2018) ensured that external feedback provided triangulation of the data (contradicting versus supporting).

In fact, the external feedback supported her personal understanding of the overall quality of the project, and that thought by other people, for the solely purpose of being as realistic as possible with regard to the artistic outcomes of the project and its legitimacy in the research field of musical expertise. Overall, she was confident in the quality of her performances, particularly from the fourth concert, and enjoyed pushing herself to work hard and collect the data. This project included music close to her heart that she wanted to perform at a higher level than previously through the use of self-regulation materials and a better understanding of how human motivation functions in her particular case. In fact, she perceived that engaging in self-regulation made her feel more efficient in her practice and experiencing progressively more flow while practicing and performing. This led her to accomplish a number of short- and long-term goals, as has been reported in other research using music students (Miksza and Tan, 2015).

Our results can also be related to the fulfillment of the cellist’s psychological needs. This aligns with previous research in which such satisfaction of needs has been related to autonomous motivation (in music, Evans and Bonneville-Roussy, 2016; in other educational contexts, Standage et al., 2005; Niemiec et al., 2006). However, the negative experiences described when playing with one of the pianists of the project was clearly a demotivator for her. In this regard, a supportive environment—from both other pianists and the praise from experts, public and critics—has been associated with better well-being (in healthcare. Deci and Ryan, 1987; Ferrand et al., 2014; in music education, Krause et al., 2019) and engaging in SRL led the cellist to take action and change this situation. This led to higher levels of enjoyment, engagement, performance, and self-determined motivation, as well as higher perceptions of musical competence—all aspects that could be understood as educational implications to better support students in the fulfillment of their basic psychological needs (Ryan and Deci, 2019).

This study has a number of methodological implications. It documents an innovative research intervention and analysis not employed before in self-regulation studies in music: intentional self-regulation and time-series analysis in music by a musician-researcher who was researching herself. This is also one of the first times that Leximancer analysis has been employed in a music study. Thus, the current study expands the positivist and post-positivist research paradigms (Denzin and Lincoln, 2000) common to the literature on self-regulation and self-determination theories. The study also contributes to the enhancement of more realist, positivist approaches to the field of artistic research where artists research themselves by rigorously merging the eternal divide of theory and practice through a multiplicity of research methods and domain-specific expertise (artistic, psychological). This combined approach is based on the assumption that:

“[…] there is no single external reality or, if there is, then it can never be fully and completely defined—as human nature is to view it through a complex set of pre-existing beliefs and experience. Phenomena, therefore, exist as conceptualized by different people. Different researchers, or even the same researcher on different occasions, can apprehend them in different but equally valid ways. Subjectivity is inherent to qualitative research and, if deliberatively applied to qualitative analysis, an asset rather than a problem. Rather than proving the existence of phenomena and measuring how those phenomena vary, qualitative research provides rich representations of phenomena” [in line with Crotty (1998) as mentioned in Yardley et al., 2020, pp. 413–414].

In addition, we are aware that there are other motivational aspects that affect a musician’s practice. Because of this, our ongoing research plans to target the role of academic emotions (as enumerated by Pekrun et al., 2002), quantity and quality of practice (following Ericsson et al., 1993), and the use of learning conditions, processes and results (in line with the model proposed by Pozo et al., 2020) during her practice. Such work will provide a holistic picture of the psychological aspects behind the practice of expert musicians.

We recognize that the study reported here has certain limitations. First, it is restricted to a sample of a single cellist and therefore the results obtained are difficult to generalize to other professional musicians. However, the implications of the use of these reflective materials on the increase of intrinsic motivation and decrease of extrinsic motivation in the cellist across time could be replicated in other longitudinal studies of professional musicians within the classical genre and those of other genres that strive for such outcomes. In this regard, our results do not aim to be prescriptive; instead, they imply that the intra-individual change in highly experienced musicians can be partly explained by *intra-mental processes* (inside the person), and to some extent through *inter-mental processes* (between people), as defined by Vygotsky (1978).

Future studies might explore self-determination within chamber music making to consider simultaneously the types of motivational orientations of groups of musicians, given that we did not explore here the motivations of the different pianists who accompanied the cellist. In addition, according to the statistical analysis, in this study we used the concerts (including all study sessions) instead of per 10 sessions to maximize significance power. Thus, high significance of the data is needed because the regressions in this particular case do not measure the independence of data collection (estimates), so the variability are, in fact, underestimates of the correct variability.

## Data Availability Statement

A selection of the datasets generated for this study are available on request to the corresponding author.

## Ethics Statement

This study involves human participants and was reviewed and approved by the Ethics Committee at the University of the Arts Helsinki, Finland. The participants provided their written informed consent to participate in this study. Written informed consent was obtained from the individuals for the publication of any potentially identifiable images or data derived from this research.

## Author Contributions

GL-Í conceived the presented idea, planned the design of the study, carried out the experiment and artistic project, collected, transcribed, and translated the data, performed the statistical and lexicometrical analyses, and took the lead in writing the manuscript with support from GM in all sections. GM developed the theory in music studies, devised the conceptual ideas of the manuscript, supervised the findings and statistical analysis of this work, contributed to the interpretation and presentation of results, and provided language support, literature advice, and writing encouragement to GL-Í. GL-Í and GM provided critical feedback, discussed the results, and helped shape the final manuscript.

## Conflict of Interest

The authors declare that the research was conducted in the absence of any commercial or financial relationships that could be construed as a potential conflict of interest.

## Footnotes

1 Amotivation has not been included in these results because the cellist never assigned a response for it.
