# Supplementary material for: Applying Self-Regulated Learning and Self-Determination Theory to Optimize the Performance of a Concert Cellist
Source: Front Psychol. 2020 Mar 6;11:385. doi: 10.3389/fpsyg.2020.00385 (PMC7067924; doi:10.3389/fpsyg.2020.00385)
Supplement: Supplementary file 2 [file Data_Sheet_2.pdf]

Appendix 2 – Feedback from critics[1] and audience

Mendelssohn CD review: Concerto: das Magazin für Alte Musik, Germany (15.1.2020), review by Peter Sühring: *"In the D-major Sonata both musicians not only meet the enormous technical requirements bordering on virtuoso. In addition to the absolutely necessary technical ability, there is also a joy of playing, with which this work achieves an adequate, particularly effective result—provocative like it was at the time. One feels aligned with one of the first critics of this work on being a 'lovely random walk on the unlimited ocean of thought', and feels here in particular the song-like rhapsodic character of almost all Mendelssohn's compositions. In the Variations Concertantes, the opulent elaborated piano part played by Olga makes again and again room for the virtuosic ways of playing by Guadalupe on the cello [...] in the coda the enharmonic confusion is played with a particular sensual gesture and sound by Guadalupe aimed at bringing such debauchery back to the basic key and original mindset. In the Albumblatt there is a romantic game by the musicians, the sound is very francophile and, attracted by the cello, one almost waits for Saint-Saëns' Swan to turn around the corner. The two instrumentalists literally break a lance for Mendelsohn's B-flat major Sonata, with a clear bubbly and melodious demanding piano part and intensive dialogic interplay [...] the music here alone is sufficiently spun—'beautiful, clear and peculiar'— exactly as one of his first listeners, Robert Schumann, would have loved."*

Mendelssohn CD review: Melómano – Classical music magazine, Spain (1.5.2019), review by Abelardo Martín Ruiz gives 5 stars ★★★★★ and monthly recommendation: *"The cellist offers a contrasting proposal through an understanding of this music from a deep sensitivity and romantic expressiveness, with the brilliance and luminosity of the author's language – a characteristic combination delimited by a resonance achieved through a complete understanding of gut strings' acoustic production. Particularly moving are the interpretations of the slow movements [...] The rapid movements possess the energy of Mendelssohn's exuberance [...] an idiomatic, prodigious knowledge of both instruments… an expressiveness of intense emotional component."*

Mendelssohn CD review: Fanfare – Classical CD review magazine, USA (1.11.2019), review by Scott Noriega: *"How does this duo sound when they perform music? As two friends having the most fun that anyone could while playing music together! From the energetic opening of the D-Major Second Sonata, through its quirky Scherzo, its Bachian slow movement, and its joyous finale, there is never a moment that the two do not give the most of themselves. The Variations Concertantes are also filled with a true sense of discovery and risk taking – just witness the breakneck speed taken in the middle variations! But my favourite work in the recital must be the B-Major Sonata. Perhaps it is the work's peculiarities which most impress on this recital: the strange ominous opening with its winding figurations, the odd back and forth between the cello and the piano in the Andante, and the jubilant Finale with its quite closing measures, music which seems to fade into the silence which surrounds it. It is in these moments when the two artists' conceptions are most realized."*

Mendelssohn CD review: Early Music Review – International review website (11.5.2019), review by Dr. James Ross: *"From her programme note, it is clear that Spanish cellist Guadalupe loves Mendelssohn's music, and perhaps even the man himself, and in that she is in complete agreement with my own tastes. Her 1725 Claude Pieray cello has been set up in the manner of early 19th-century instruments and fairly sings Mendelssohn's lovely lyrical lines, while Olga's virtuosic and passionate playing on her 1862 Erard piano is also wonderfully expressive. [...] The energy and technical assurance of all of this music is a testimony to the genius of its remarkable composer."*

Mendelssohn CD review: Musicalifeiten – Classical music website, The Netherlands (1.5.2019), review by Jan De Kruijff: *"Perhaps this new recording [has] a bit more of the [original] spirit [than previous recordings by other artists]. As a first step towards "authenticity", the duo provides very spiritual interpretations in which the piano sparkles and the cello can be heard warm and full of brilliance. Just listen to what the two make of the second part of the Sonata no. 1, which is at the same time scherzo and slow—well characterised—or how nice are the Variations Concertantes by presenting a mixture of panache and subtlety. The "corner" parts of Sonata no. 2 also sound very spontaneous."*

Mendelssohn CD review: BBC Music Magazine – Classical music magazine UK (1.4.2019), review: *"Mendelssohn's cello works call for spirited playing, which is what we get here. The Érard piano sparkles; the cello playing is bold and confident."*

Mendelssohn CD review: Classique HD – Independent Review Site, France (3.4.2019), review by Nicolas Roberge: *"When you look carefully through the sound, you can guess the fluid and sensitive playing of Guadalupe, the notes are extremely sensitive and full of poetry. Olga's piano is doing its job, its accompaniment is in place*

[1] Note: reviews have been translated into English when appropriate, and only aspects related to the playing are included (we have removed, for example, any descriptions on compositional aspects).

and the flexibility of the wrist makes Guadalupe's playing even more divine. [...] We reach a fullness in «Assai Tranquillo in B minor», it is beautifully interpreted."

Mendelssohn CD review: Pizzicato – Music journal, Luxembourg (14.3.2019), Mendelssohn CD review by Uwe Krusch gives 5 stars ★★★★★: "*Guadalupe and Olga play Mendelssohn's cello chamber works on period instruments and orientate themselves towards historical sources. But since they are spicing their performances with own ideas, the result is rhetoric and therefore really gripping."*

Mendelssohn CD review: Rondo Classic – Music magazine, Finland (1.3.2019), Mendelssohn CD review by Antti Häyrynen: *"[...] for example, in the D major Sonata op. 45 both skill challenges and romantic expressions come out with impressive results."*

Concert 1, audience feedback: *Thank you so much for the beautiful evening!*

Concert 1, audience feedback: *I loved the display of historical instruments, and the understanding of the repertoire was unique, thanks a lot.*

Concert 2, audience feedback: *Thank you for the wonderful concert you gave, I am looking forward to your future visits.*

Concert 2, critic: Muusika – Classical Music Magazine, Estonia (2.2.2017), Concert review by Aleksandra Dolgopolova: *"An ideal ensemble. The musicians felt each other like Siamese twins [...] genuine feelings and choices, and a slightly extravagant performance."*

Concert 3, critic: Savon Sanomat – Regional Newspaper, Finland (27.5.2017), Concert review by Jussi Mattila: *"Much of this warm-hearted, gentle and social side of Beethoven was emphasized in the concert [...] the performances breathed together and were characterized by personal touches and virtuosity, with an emphasis on colourful expression. Cellist López-Íñiguez's performance was filled with lively emotions and a rich palette of colours, finding expressiveness especially in the low and middle registers of the classical cello."*

Concert 4, audience feedback: *It was a lovely concert, well done both of you!*

Concert 5, audience feedback: *It was a wonderful concert in so many ways, especially bringing us back to the past when Mendelssohn was alive with your instruments and ways of playing, thank you for this!*

Concert 5, audience feedback: *Thank you for this evening's wonderful concert. It was very touching and intelligent playing. We liked the lecture part as well, this information was new to us, although we are not newcomers in the classical music. God bless your talent!*

Concert 6, audience feedback: *I thank you for your excellent participation in our events this spring. Your presentation on interpretation of Beethoven's early cello works on period instruments was so enlightening and fit perfectly into the theme of our featured exhibit. We were thrilled to have a full house for your concert on our 1823 Broadwood fortepiano and appreciate your generosity in sharing your musical talents with our followers.*

Concert 6, critic: Campbell Express – American Newspaper, San José, California (25.4.2018), Concert review by Guy Takamatsu: *"Dr. Mok and Dr. López-Íñiguez were able to generate beautiful music. Performances on these old instruments gave the audience a feel for how the music sounded in 1796. The concert was a delightful and informative experience."*

Concert 8, audience feedback: *This was definitely my favourite concert in the entire festival. The chamber musicianship was incredible, and the music so lovely and touching. You both were very elegant in your playing and your attitude. Thanks a lot!*

Concert 9, audience feedback: *You both are virtuosos, and this evening was most delightful. The sound of the instruments was magical, and the music making brought me to a 19th century home concert. Many thanks for coming here!*
