# Supplementary material for: Applying Self-Regulated Learning and Self-Determination Theory to Optimize the Performance of a Concert Cellist
Source: Front Psychol. 2020 Mar 6;11:385. doi: 10.3389/fpsyg.2020.00385 (PMC7067924; doi:10.3389/fpsyg.2020.00385)
Supplement: Supplementary file 3 [file Data_Sheet_3.pdf]

Appendix 3 – Expert feedback

Year	Event Day and Place	Feedback
2016	4 October / *Sellosali* Concert Series, Espoo (Finland)	This concert left mixed feelings. The musical and artistic ideas, knowledge of the HIP, as well as the passion of the music were well presented, but the playing of the cellist was sometimes lacking full technical control. Most of the time the intonation was excellent but sometimes there were mistakes even in fairly easy passages due to exhaustion – though playing using different tunings, keyboards and cello bows is something that makes even the most famous players struggle, so the cellist was really brave and this was also an insightful aspect for this concert. The pianist played well but she was lacking an interaction with the cellist and passion to match the demands of the music, although the duo played well together, partly thanks to the excellent chamber musicianship of the cellist.
	1 November / *Kloostri Ait*, Tallinn (Estonia) & broadcasted by Klassikaraadio in Estonia	I listened to the radio broadcast of this concert. It was a totally different level both regarding emotional/musical qualities and the technical level of the cellist, which is not an easy thing considering the mics sound relatively close to the cello, so she did excellent work controlling herself. The sound balance between the cello and the piano was good, and the pianist was playing more expressively. Generally, the accuracy was better than in the previous concert, and the tone quality excellent.
2017	24 May / *Nordic Historical Keyboard Festival*, Kuopio (Finland)	I watched a live video recording of this concert. There were several issues regarding concentration for both the pianist and the cellist. The atmosphere and style of the music, and the contrast of dynamics was rather clear, but for some reason both musicians were holding back and underplaying if we consider their abilities.
	12 September / *Aino Ackté Festival*, Helsinki (Finland)	This concert left mixed feelings although it was clearly better when the previous concerts. The style, knowledge and passion were again carried well, but the playing of the cellist was lacking full control a few times, possible due to concentration issues, I think. Most of the time the intonation was excellent but sometimes there were mistakes in fairly easy moments which leads to the conclusion of tiredness of some sort. The pianist played well but she was lacking an interaction with the cellist and she could not match the cellist's passion according to the demands of the music. Most of the time she also underplayed causing then a problem of balance between the instruments.
	28 November / *Uusi Paviljonki* Concert Series, Kauniainen (Finland)	All artistic aspects of the music were this time presented beautifully by both musicians, and the qualities of the cellist's playing remained in a high and even level during the whole concert. The cello sound was exceptionally good and it has improved a lot. The pianist played brilliantly too, but perhaps her skills and experience are focused to solo playing and consequently the cellist had to, once again, rely on her exceptional chamber musicianship. Luckily the cellist was very well able to follow her when needed, but also communicating strongly enough to get her musical ideas through to the pianist.
20 18	18 April / *Beethoven Research*	I heard a tape of this concert. Very interesting piano partner, with a beautiful tone, and possibly the best chamber musician for the cellist to date. Musical ideas were much better than before, most likely due to the chamber support the

	Center, San José, California (USA)	cellist got from her. The cello was robust and confident, and Beethoven seems to be a natural language for her. The piano and cello balance was perfect, and the cellist was obviously inspired with new ideas after her musicological research there, as I could notice she took some personal, creative licenses and musical risks.
	15 July / *Piano Salon Christophori*, Berlin (Germany)	This concert was very interesting. The acoustics were terrible for this kind of instruments, so the cellist and pianist had difficulties to connect, and maybe that led to some tempo and rubato disagreements. It was clear they both were tired by the end of the concert because this is a very long and heavy program, and the piano volume was too robust for the gut strings of the cello, so she really had to work hard to bring out the most of the instrument, which is a pity. No problems technically or musically otherwise, I believe the work left for the cellist is to polish and polish the small details and keep in mind that the pianist is a very robust player and so are the pianos sometimes.
	8 August / *BRQ Vantaa Festival*, Vantaa (Finland)	This was for sure the best performance I have heard from the cellist so far. Musical and artistic ideas, knowledge of the HIP as well as passion of the music were beautifully executed, and the qualities of her playing remained in a high and even level during the whole concert. The cello sound was again exceptionally beautiful. The piano player played brilliantly, but my impression is that she has not much improved as a chamber music partner, though the aesthetic ideas by the cellist seem to have influenced her on the use of pedal, articulation and timing, for example. Luckily the cellist was very well able to follow her when needed as usual, but also communicating strongly enough to get her musical ideas through her playing – a good example of her strong musical identity and personality. The feedback of the audience was enthusiastic and warm.
	25 October / *Philharmonic Society's Small Hall*, Moscow (Russia)	I watched a video of this concert and the first thing I noticed was the enthusiastic response of the audience throughout, which is a milestone for the cellist who was playing first time in a country where they have possible the very best cellists on earth. The chamber musicianship was at its best if I think backwards for this whole project. The cellist was faultless with her technic and expression, and so was the pianist. A pleasure to see the development of the duo and, particularly, the musical growth of the cellist who could play now in the league of international stars if given the resources and opportunities she deserves.
	Recording, Kauniainen, Finland, 16-17 August and 1-2 September	The recording came out very well. It differs clearly from other recordings available by having lively tempos which follow the ideas of the composer, and because there is much more passion. The special musical expressions and cello technical solutions are following HIP, much more than any other specialist to date in my opinion. The pianist plays brilliantly but she could have used more rubato when possible as well as separations of both hands (bass notes should come first). The sound engineering didn't succeed so well, and the result is maybe not as beautiful as the live performances of this duo, yet it is a high-quality recording of a highly demanding program for period instruments (and gut strings for the cellist!), only possible for virtuosos on such instruments. My impression is that the pieces which were performed more often, like the D major Sonata, came out the best, but some other listeners and international radios and critics seem to enjoy particularly the other pieces.
