# Supplementary material for: Applying Self-Regulated Learning and Self-Determination Theory to Optimize the Performance of a Concert Cellist
Source: Front Psychol. 2020 Mar 6;11:385. doi: 10.3389/fpsyg.2020.00385 (PMC7067924; doi:10.3389/fpsyg.2020.00385)
Supplement: Supplementary file 4 [file Data_Sheet_4.pdf]

Appendix 4 – Scores for the five types of motivation

	Event	Average score pre-practice	Minimum score pre-practice	Maximum score pre-practice	Average score post-practice	Minimum score post-practice	Maximum score post-practice
Intrinsic Motivation	1	6.4	5	9	7.7	6	10
	2	6.3	5	9	7.8	6	10
	3	6.6	5	9	6.9	6	10
	4	7.1	6	9	7.2	5	9
	5	7.7	7	9	8	6	9
	6	7.5	6	8	8.2	7	9
	7	7.9	7	9	8.3	7	10
	8	8	7	9	8.6	7	10
	9	7.9	7	9	8.4	7	10
	10	8.8	8	10	9.2	8	10
Integrated Regulation	1	0,9	0	2	1,3	0	2
	2	0.7	0	2	1.1	0	2
	3	0.9	0	2	0.9	0	2
	4	1.1	0	2	1.1	0	2
	5	1.4	0	2	1.4	0	2
	6	1.3	0	2	1.5	0	2
	7	1.1	0	2	1.8	1	2
	8	1.6	1	2	1.8	1	2
	9	1.5	0	2	1.9	1	2
	10	2	2	2	1.9	1	2
Identified Regulation	1	0.7	0	2	0.9	0	2
	2	0.6	0	2	1	0	2
	3	0.5	0	2	0.9	0	2
	4	0.7	0	2	0.9	0	2
	5	1.1	0	2	1.3	0	2
	6	1.1	0	2	1.5	0	2
	7	0.9	0	2	1.6	0	2
	8	1.4	0	2	1.6	1	2
	9	1.3	0	2	1.7	1	2
	10	1.9	1	2	1.9	1	2
Introjected Regulation	1	1	0	2	1.6	0	2
	2	0.8	0	2	1.2	0	2
	3	1	0	2	1	0	2
	4	1.4	0	2	1.4	0	2
	5	1.6	0	2	1.6	0	2
	6	1.5	0	2	1.8	0	2
	7	1.2	0	2	2	2	2
	8	2	2	2	2	2	2
	9	1.9	1	2	2	2	2
	10	2	2	2	2	2	2
External Regulation	1	8.5	5	10	5.7	5	8
	2	8.1	5	10	5.6	5	7
	3	7.4	5	8	6.8	5	8
	4	7.0	5	9	6.0	5	9
	5	6.8	5	9	6.0	5	7
	6	6.4	5	7	5.7	5	7
	7	6.1	5	7	5.5	5	7
	8	5.8	5	7	5.2	5	6
	9	5.6	5	6	5.1	5	6
	10	5.2	5	6	5.0	5	5
